# Medulloblastoma response to mevalonate pathway inhibition is independent of p53 status

**DOI:** 10.1186/s13062-026-00765-9

**Published:** 2026-04-01

**Authors:** Charley Comer, Christopher Edwards, Sabrina Caporali, Yuhui Lu, Alessio Butera, David Michod, Andreas J. Gruber, Ivano Amelio, Maria Victoria Niklison-Chirou

**Affiliations:** 1 Life Sciences Department, University of Bath, Claverton Down, Bath, BA2 7AY UK; 2 Chair for Systems Toxicology, University of Konstanz, Konstanz, Germany; 3 Cancer Section, Development Biology and Cancer Programme, UCL Great Ormond Street Institute of Child Health, London, UK; 4 Applied Bioinformatic Laboratory, University of Konstanz, Konstanz, Germany; 5 Blizard Institute, Barts and the London School of Medicine and Dentistry, Queen Mary University of London, London, UK

**Keywords:** p53, Mutant p53, Cholesterol, Medulloblastoma, Metabolism

## Abstract

Medulloblastoma (MB) is the most common primary solid paediatric brain tumour. It arises in the cerebellum, the part of the brain responsible for equilibrium and coordination. Molecular profiling has identified four MB subgroups named Wingless (WNT), Sonic hedgehog (SHH), Group 3 (G3), and Group 4 (G4), each of which possesses distinct genetic backgrounds and clinical outcomes. The treatment of MB requires intensive multimodal therapy that is associated with significant long-term side effects. Despite this necessary aggressive approach, MB remains fatal in approximately 30% of patients due to tumour recurrence and/or metastatic spread to the spinal cord. Thus, there is an urgent need for novel, less toxic and effective therapies for MB. The tumour-suppressor p53 is mutated in up to 30% of SHH-MB tumours and is associated with significantly poorer patient outcomes. Mutations in p53 have been acknowledged to confer oncogenic “gain-of-function” properties, including activation of the mevalonate pathway (MVP), which drives cholesterol and isoprenoid biosynthesis. MB is sensitive to inhibition of the MVP by treatment with simvastatin, leading us to hypothesise that targeting the mutant p53 (mutp53)-MVP axis could further sensitise mutp53 SHH-MB to statins. In this study, we show that silencing p53 does not alter simvastatin sensitivity in either mutp53 SHH-MB or wild-type p53 G3-MB cells. Moreover, p53 silencing had no effect in the mRNA expression of the key MVP enzymes 3-hydroxy-3-methylglutaryl-CoA reductase (*HMGCR*), mevalonate kinase (*MVK*), mevalonate decarboxylase (*MVD*), and farnesyl diphosphate synthase (*FDPS*). Silencing p53 also failed to affect simvastatin-induced cell cycle arrest or impact the sensitivity of MB cells treated with simvastatin in combination with hypoxia, X-ray or azacitidine, an epigenetic therapy with DNA methyltransferase inhibition. Collectively, our findings indicate that MVP function is independent of p53 in MB.

**Supplementary Information:**

The online version contains supplementary material available at 10.1186/s13062-026-00765-9.

## Introduction

Brain tumours are the leading cause of cancer-related death in children [1]. The most common (~20% of cases) malignant paediatric brain tumour is medulloblastoma (MB), an embryonal tumour that arises in the cerebellum [2]. Treatment of MB requires aggressive multimodal therapy, including maximal safe resection, chemotherapy and craniospinal irradiation, which is associated with substantial toxicity. Consequently, most survivors experience long-term side effects, such as learning and memory impairments, that significantly affect their quality of life [3, 4]. In the last decade, transcriptional profiling has revealed four heterogeneous subgroups in MB named Wingless (WNT, good prognosis), Sonic hedgehog (SHH, intermediate prognosis), Group 3 (G3, poor prognosis), and Group 4 (G4, intermediate prognosis), each with distinct demographics, genetic alterations, and clinical outcomes [5–7]. While personalised treatment strategies tailored for WNT and SHH subgroups have improved survival outcomes or reduced side effects, MB remains fatal for ~30% of patients as a result of tumour recurrence and/or metastasis to the spinal cord [3]. Thus, there is an urgent need for novel, less toxic, effective therapies for MB.

**Fig. 1:**
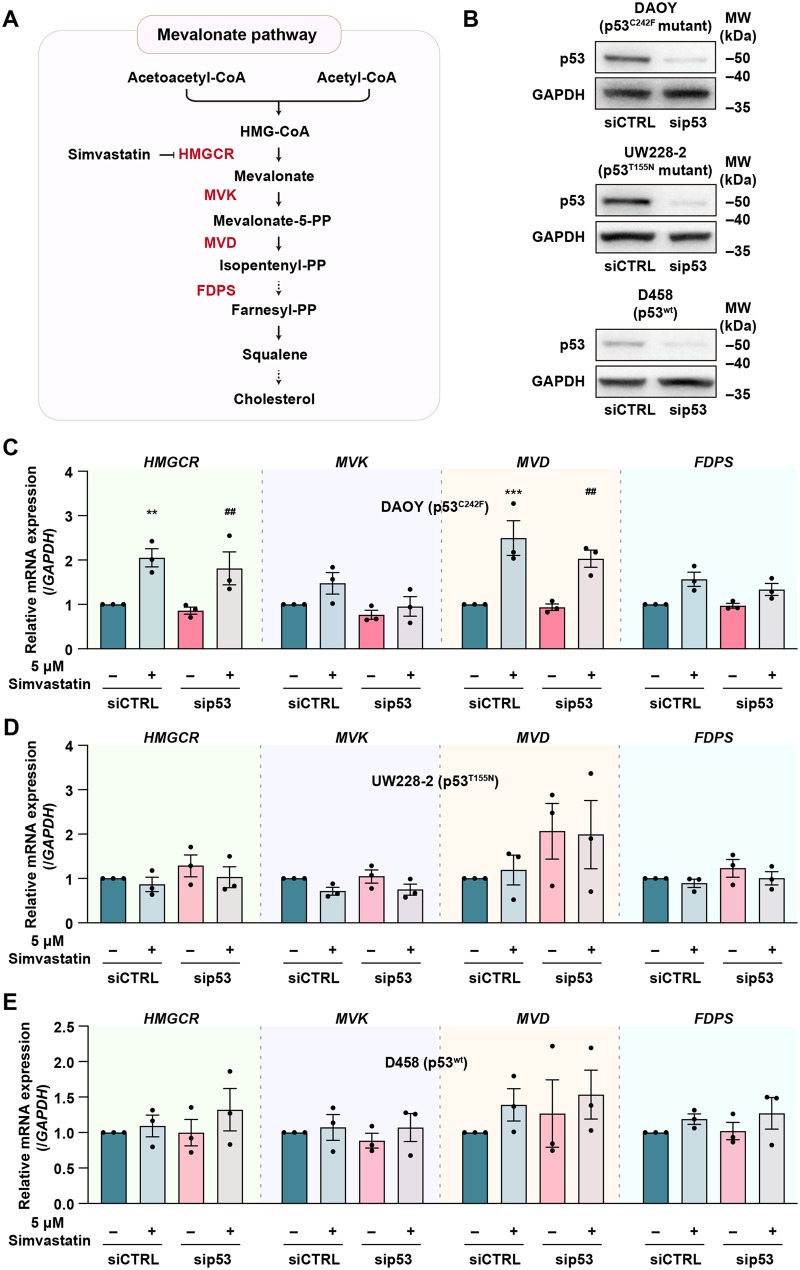
Mevalonate pathway gene expression in MB cells upon simvastatin treatment. **A**) Schematic chart of MVP resulting in cholesterol biosynthesis. Important MVP enzymes are highlighted in red, with dashed arrows representing multi-enzyme processes. **B**) Representative immunoblot showing *TP53* silencing in DAOY, UW228-2 and D458 cells. **C–E**) RT-qPCR analysis of *HMGCR*, *MVK*, *MVD*, and *FDPS* mRNA expression in DAOY (**C**), UW228-2 (**D**) and D458 (**E**) cells. TATA-box binding protein (*TBP*) gene was used as a control. A one-way ANOVA with multiple comparisons was performed (*n* = 3). **p < 0.01, ***p < 0.001 vs. the siCTRL untreated group; ^##^*p* < 0.01 vs. the sip53 untreated group.

One potential strategy to meet this need involves targeting abnormal metabolic processes unique to cancer cells that play a key role in promoting their growth, survival and/or spread. Among these, tumour reprogramming of cholesterol metabolism has drawn significant interest [8, 9], particularly in the context of brain tumours which reside in the body’s most cholesterol-dense tissue [10]. *De novo* biosynthesis of cholesterol occurs in the endoplasmic reticulum (ER) through the mevalonate pathway (MVP), which also generates intermediate isoprenoids such as farnesyl pyrophosphate (farnesyl-PP) and geranylgeranyl pyrophosphate (Fig. 1A) required for the post-translational localisation/activation of various cell cycle regulatory or cell migration proteins (e.g. Ras, Rac, and Rho GTPases) [11–14]. To maintain cholesterol homeostasis, the MVP is subject to strict feedback control whereby levels of ER membrane cholesterol dictate activation of the master regulatory transcription factor Sterol Regulatory Element (SRE)-Binding Protein-2 (SREBP-2). MVP flux is increased following sensing of cell sterol depletion through the ER-membrane release of SREBP-2, which subsequently undergoes proteolytic cleavage and translocates to the nucleus to activate transcription of MVP enzymes by binding SREs within their gene promoter regions. SREBP-2 remains ER-bound when cholesterol levels are sufficient, preventing its activation and thereby limiting MVP flux [15, 16]. Aberrant SREBP-2 activation has been observed in multiple cancers, with dysfunction of the tumour suppressor p53 playing a pivotal role [16].

As the “guardian of the genome”, p53’s tumour-suppressive function was first largely attributed to its ability to halt the proliferation of cells with damaged or mutated DNA by transcriptionally activating genes involved in DNA repair, cell cycle arrest and apoptosis. However, p53 biology is now recognised to be far more complex, with regulation of various “non-canonical” pathways such as metabolism, senescence and autophagy also implicated within its tumour-suppressive activity [17–20]. More than half of all human cancers exhibit mutations in the *TP53* gene that can generate neomorphic p53 proteins. These mutations, which typically affect amino acids within the DNA-binding domain, not only result in loss of function but also confer novel gain-of-function properties, or, in some cases, preserve residual activities of the wild-type p53 (wtp53) protein, that can drive malignant progression [21–23].

Extensive investigation of the significance of mutant p53 (mutp53) proteins has led to the identification of regulations of the cancer cell metabolism [24, 25]. In a landmark study, Freed-Pastor et al. first reported that mutp53 contributes to the malignant morphology of breast cancer cells through binding SREs within the gene promoter regions of MVP enzymes to support SREBP-2-dependent upregulation of their expression [26]. Loss of wtp53 function also impairs expression of the cholesterol transporter ATP-binding cassette transporter 1 (ABCA1), leading to depletion of ER cholesterol and rapid SREBP-2 maturation that ultimately facilitates liver tumour development [27]. In pancreatic ductal adenocarcinoma (PDAC), mutp53 further disrupts MVP feedback inhibition and protects against lipotoxicity by upregulating expression of acetyl-CoA acetyltransferase 1 (ACAT1), also known as SOAT1, to esterify cholesterol for lipid droplet storage [28]. Mutp53 upregulation of isoprenylcysteine carboxyl methyltransferase (ICMT), which catalyses final step of protein prenylation, has likewise been observed across multiple cancers. In turn, the MVP can also protect mutp53 from ubiquitination via the production of mevalonate-5-phosphate (mevalonate-5-PP) [29, 30] and geranylgeranylation of Rho-A [31]. This culminates in a positive-feedback loop whereby mutp53 upregulates MVP to maintain its own stability, leaving tumours driven by mutp53 potentially vulnerable to MVP inhibition [16, 30].

Statins are widely used to lower blood cholesterol levels for prevention of cardiovascular disease by competitively inhibiting 3-hydroxy-3-methylglutaryl-CoA reductase (HMGCR), the rate-limiting enzyme of the MVP. We have recently shown HMGCR to be upregulated in MB and that simvastatin, which can cross the blood-brain barrier, inhibits MB cell migration and growth both in vitro and in vivo [32]. With *TP53* mutated in up to 30% of SHH-MB tumours and associated with significantly poorer prognosis [33, 34], we next chose to investigate whether targeting the mutp53-MVP axis could further sensitise mutp53 SHH-MB to statins. By assessing the impact of *TP53* depletion on statin sensitivity and MVP gene expression in mutp53 SHH and wtp53 MB cell lines, we found that neither wtp53 nor mutp53 regulates the MVP in these cells, suggesting that alternative factors may govern this pathway in MB.

## Results

### Mevalonate pathway gene expression in MB is independent of p53 proteins

To investigate the relationship between p53 status and the susceptibility of MB to MVP inhibition, we first examined whether p53 influences the expression of key metabolic enzymes (Fig. 1A) in G3-MB cell line D458, which carries wtp53, and SHH-MB cell lines DAOY and UW228-2, which harbour p53^C242F^ mutant and p53^T155N^ mutations, respectively [35]. We examined whether *TP53* knockdown (*TP53*-KD) by siRNA transfection alters the mRNA expression of *HMGCR*, mevalonate kinase (*MVK*), mevalonate decarboxylase (*MVD*) and farnesyl diphosphate synthase (*FDPS*), enzymes of the MVP [36]. Successful KD was confirmed by measuring p53 protein levels in both scramble-siRNA (siCTRL) and *TP53-*siRNA (sip53) transfected groups (Fig. 1B). Comparative analysis of p53 protein level among the different cell lines, confirmed the expected increased stability of mutp53 compared to the wtp53 (Supplementary Fig. 1A). A decrease in *CDKN1A* (p21 gene) mRNA level was observed following *TP53*-KD in UW228-2 and D458 confirming effective depletion of wtp53 (Supplementary Fig. 1B). DAOY on the other hand exhibited increased *CDKN1A* mRNA expression following *TP53*-KD, which may reflect a direct or indirect impact of mutp53 on p21 expression level (Supplementary Fig. 1B). We further posited that the p53-family member p73 could be enhanced as part of a compensatory response following loss of p53. In particular, we tested the isoform *TAp73*, which can promote p21 transcription, shares high homology with p53 and plays a role in governing MB metabolism [37–39]. As shown in Supplementary Fig. 1C, the mRNA levels of *TAp73* were unchanged or marginally changed following *TP53*-KD, ruling out its compensatory induction.

Next, we conducted real-time quantitative PCR (RT-qPCR) analysis, which revealed that *TP53* depletion showed no significant effect on transcription of MVP enzymes in any of MB cell lines tested (Fig. 1B–E). MVP enzyme expression was also evaluated in these cells following treatment with MVP inhibitor (simvastatin, 5 μM) for 24 hours. Interestingly, simvastatin treatment significantly increased MVP enzyme expression in control and *TP53*-KD DAOY cells (Fig. 1C), while no effect was observed in UW228-2 and D458 (Fig. 1D, E). Taken together, these findings indicate that while simvastatin-induced upregulation of MVP enzymes is more pronounced in certain SHH p53-mutant MB cell lines such as DAOY, regulation of MVP enzyme expression likely occurs independently of mutp53 or wtp53 expression.

### Simvastatin effects on cell cycle are independent of p53 in MB

The p53 protein plays a crucial role in cell cycle regulation by inducing cell cycle arrest in response to DNA damage, thereby preventing the propagation of damaged cells and maintaining genomic stability [40]. Simvastatin treatment was reported to induce cell cycle arrest in the UW228-2, D425, and ICb-1299 MB cell lines; however, the magnitude of this effect was modest [32]. Hence, here we tested whether p53 status may modulate MB cell cycle arrest induced by simvastatin (Supplementary Fig. 2A).

**Fig. 2:**
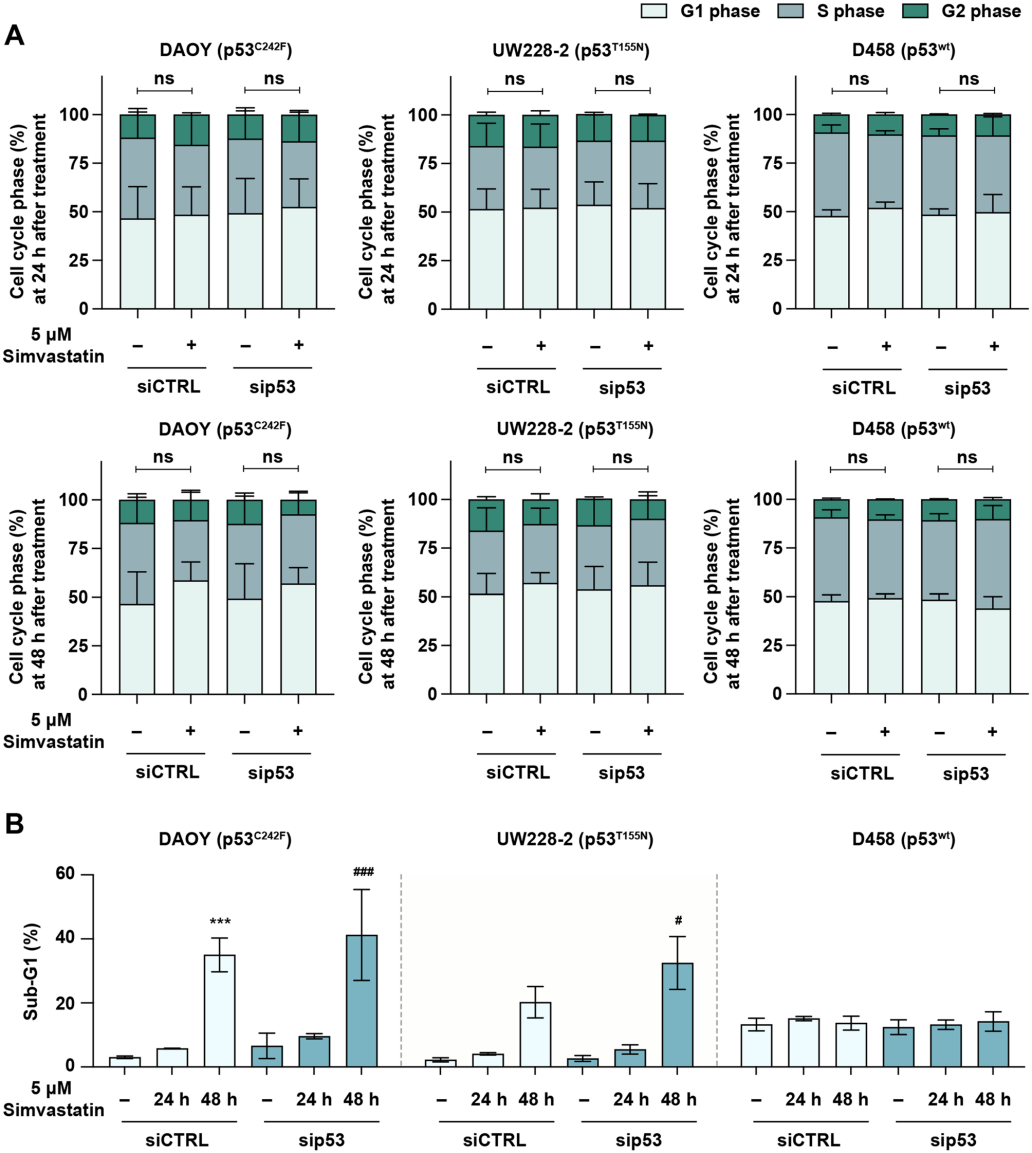
Cell cycle progression in MB cells upon simvastatin treatment. **A**) Cell cycle profile of siCTRL and sip53 MB cells treated with 5 µM simvastatin for 24 and 48 hours. **B**) Analysis of sub-G1 phase in MB cells after 24 and 48 hours of simvastatin treatment with or without *TP53* silencing. A one-way ANOVA with multiple comparisons was performed (*n* = 2). ***p < 0.001 vs. the siCTRL untreated group; ^#^*p* < 0.05, ^###^*p* < 0.001 vs. the sip53 untreated group; “ns” is not significant.

Simvastatin induced a modest G1 phase accumulation in DAOY cells at 48-hour time point, however this appeared to be not dependent on mutp53 expression, as *TP53*-KD displayed a profiled comparable to the control cells (Fig. 2A). *TP53*-KD was also found to have no overall significant effect on the cell cycle distribution of these cell lines. (Fig. 2A). These data suggest that depletion of mutp53 or wtp53 does not impact cell cycle progression in MB cells in simvastatin treatment and resting condition.

A significant increase in the sub-G1 fraction, indicative of apoptotic cells, was observed in the mutp53 MB cells lines DAOY and UW228-2 after 48 hours treatment with simvastatin. This effect was conserved across both control and *TP53*-KD DAOY and UW228-2 cells (Fig. 2B, two left panels). The wtp53-harboring D458 line on the other hand maintained consistently low sub-G1 levels across all time points and conditions (Fig. 2B, right panel). These results indicate that DAOY and UW228-2 cells, which harbour mutp53 proteins, are more sensitive to simvastatin than D458 cells carrying wtp53 proteins. However, depletion of p53 protein does not alter susceptibility to simvastatin in any of these three cell lines.

### Simvastatin affects MB cell viability independently of p53 status

**Fig. 3:**
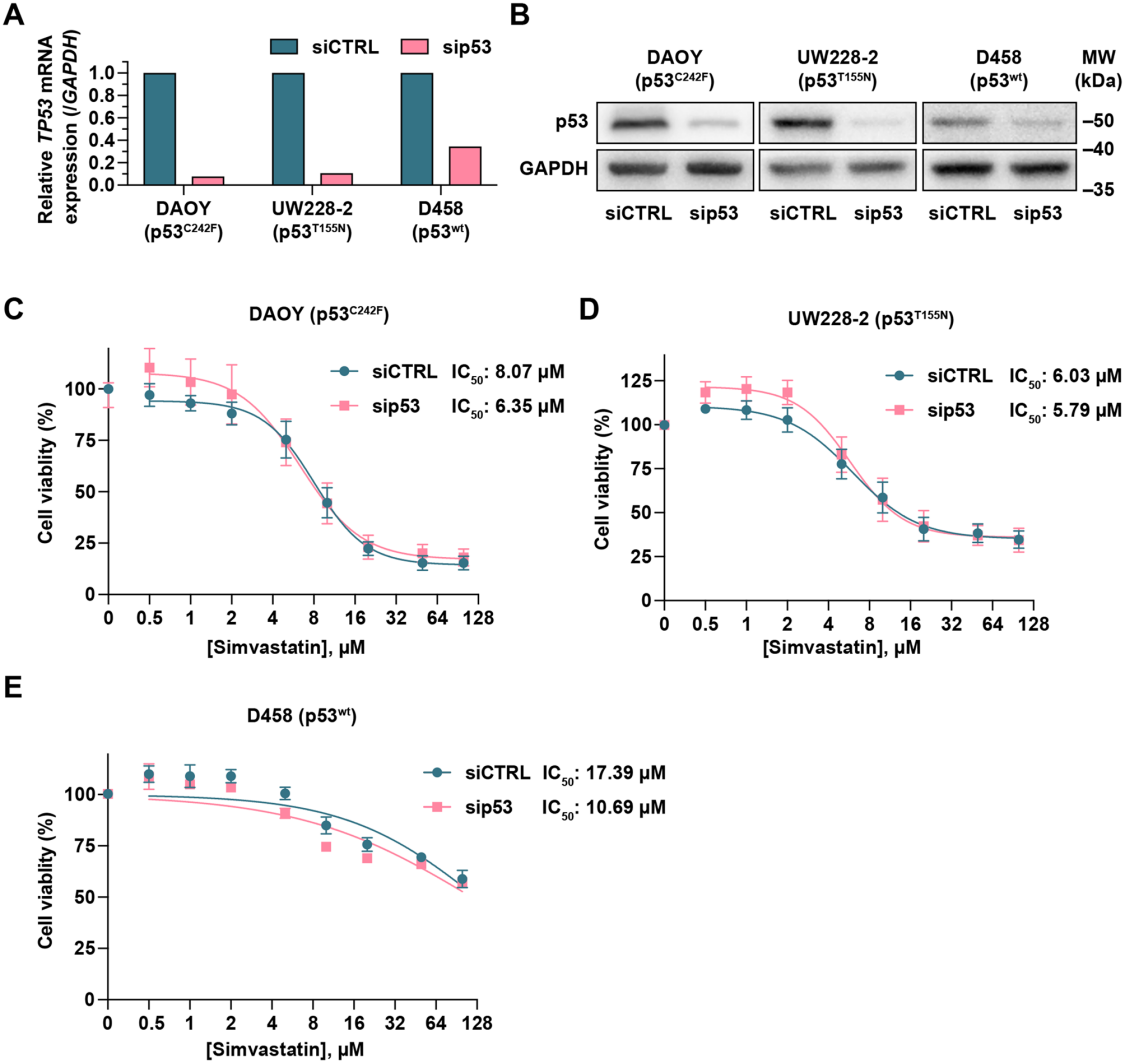
MB cell sensitivity to simvastatin upon simvastatin treatment. **A, B**) Representative RT-qPCR (**A**) and immunoblot analyses (**B**) showing p53 levels following knockdown in DAOY, UW228-2 and D458 cells. **C–E**) Dose-response curves were determined using the MTT assay in SHH-MB DAOY (**C**), UW228-2 (**D**) and G3-MB D458 (**D**) cells. MB cells were treated in the presence of increasing concentrations of simvastatin (0.5, 1, 2, 5, 10, 20, 50 and 100 µM) for 48 hours. All values were normalised to the siCTRL untreated group, which was set to 100%. Curve fitting and IC_50_ values were evaluated using GraphPad prism 10 with a nonlinear regression model ([inhibitor] vs. response, variable slope, four parameters) (*n* = 3)

To comprehensively test the impact of p53 proteins on MVP inhibitor–simvastatin sensitivity in MB, we conducted half-maximal inhibitory concentration (IC_50_) analyses in DAOY, UW228-2, and D458 proficient and deficient for the respective *TP53* mRNA and proteins (Fig. 3A, B).

All the MB cell lines show an expected reduced viability after simvastatin treatment in a dose-dependent manner (Fig. 3C–E). Mutp53-expressing DAOY and UW228-2 control cells were the most sensitive to simvastatin treatment with IC_50_ ranging between 6 and 8 µM (Fig. 3C, D). Wtp53-harboring D458 cells were the least sensitive MB cell line to simvastatin with an IC_50_ of 17.39 µM (Fig. 3E). Comparison of simvastatin IC_50_ values between *TP53*-KD cells versus controls revealed that silencing of both wtp53 and mutp53 did not significantly alter sensitivity to simvastatin in any of the MB cells (Fig. 3C–E). Collectively, these data indicate that the mutational p53 status does not influence simvastatin sensitivity in MB cells.

### Hypoxia does not affect MB response to simvastatin irrespective of p53 status

Fluctuating O_2_ availability within the tumour microenvironment frequently creates regions of hypoxia that are often associated with increased tumour aggressiveness. In hypoxic conditions, hypoxia-inducible factors (HIF) such as HIF-1-alpha (HIF-1α) escape ubiquitination-dependent degradation and subsequently accumulate to activate transcriptional programmes that promote tumour cell survival. Increased HIF-1α levels in HepG2 liver cancer cells have also been shown to drive the upregulation of *HMGCR* by enhancing its transcription [41]. Given this data and the extensive crosstalk reported between both wtp53 and mutp53 and HIF-1α [42–44], we tested whether modulating p53 protein expression under hypoxia could influence MB response to simvastatin treatment.

**Fig. 4:**
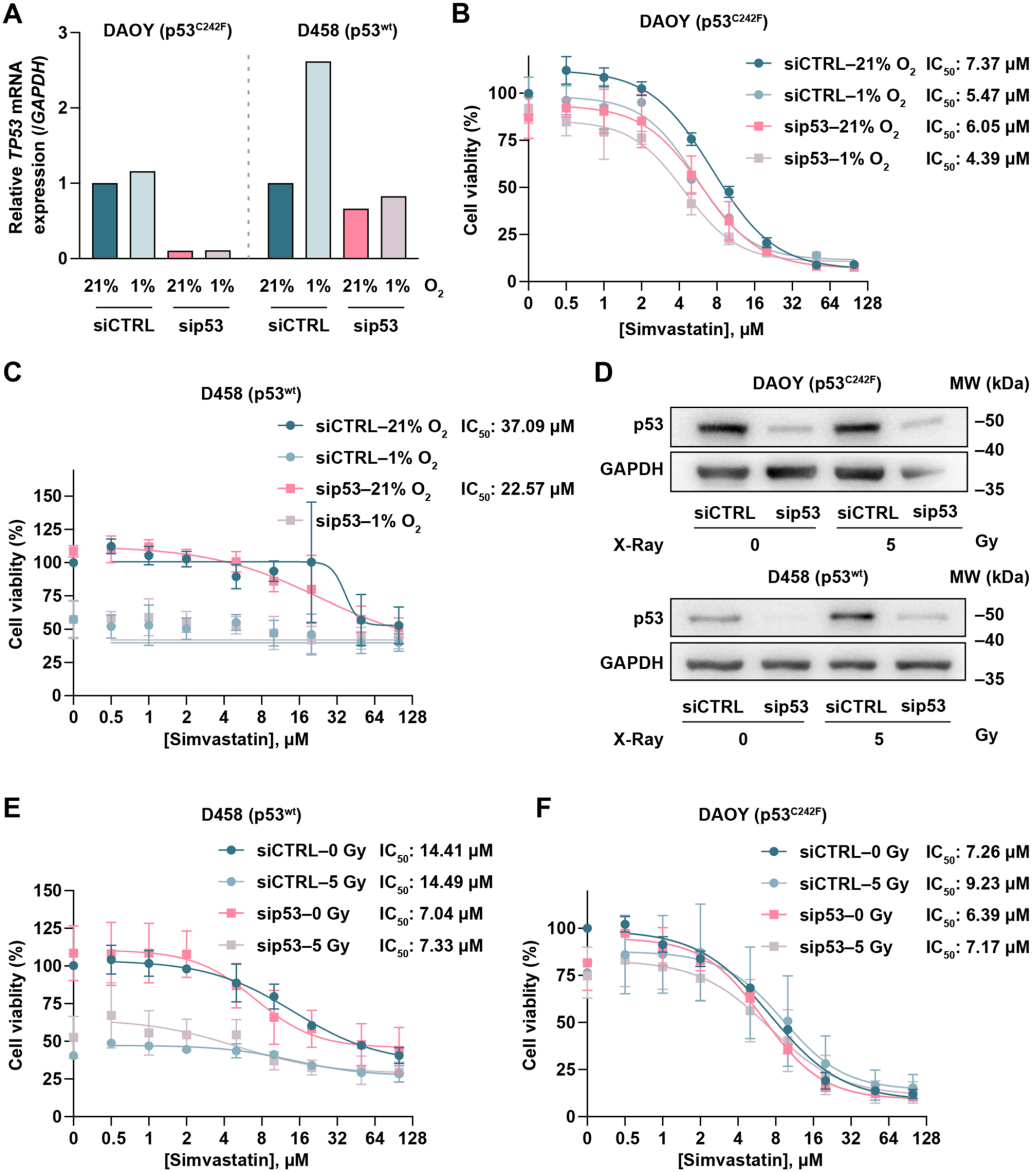
Hypoxia and DNA damage effect on simvastatin response of MB cells. **A**) Representative RT-qPCR analysis of *TP53* level in DAOY and D458 MB cells. 21% O_2_ = normoxic condition, 1% O_2_ = hypoxic condition. **B, C**) MTT analysis of siCtrl/sip53 DAOY (B) and D458 (**C**) cell viability under normoxic and hypoxic conditions following treatment with increasing concentrations of simvastatin (0.5, 1, 2, 5, 10, 20, 50 and 100 µM) for 48 hours. All values were normalised to the siCTRL untreated group, which was set to 100%. **D**) Representative immunoblot showing *TP53* silencing in DAOY and D458 cells following X-ray irradiation (5 Gy). **E, F**) Dose-response curves were determined using the MTT assay in D458 (**E**) and DAOY (**F**) cells following X-ray irradiation, with or without *TP53* silencing. MB cells were treated in the presence of increasing concentrations of simvastatin (0.5, 1, 2, 5, 10, 20, 50 and 100 µM) for 48 hours. All values were normalised to the siCTRL untreated group, which was set to 100%. Curve fitting and IC_50_ values were evaluated using GraphPad prism 10 with a nonlinear regression model ([inhibitor] vs. response, variable slope, four parameters) (*n* = 3)

To explore this, we first evaluated *TP53* mRNA *e*xpression in DAOY and D458 cells exposed to normoxia (21% O_2_) or severe hypoxia (72 hours at 1% O_2_). Hypoxia exposure had no effect on *TP53* transcription in mutp53-expressing DAOY cells while led to increased *TP53* mRNA in D458 cells (Fig. 4A). Next, we investigated the effect of hypoxia (1% O_2_) on MB cell viability and whether p53 status modulates their sensitivity to varying doses of simvastatin (0–100 µM) under this condition. DAOY cells were less sensitive to hypoxia-induced cell death, while hypoxia alone induced substantial cytotoxicity in D458, with a similar decrease to ~50% viability in both control and *TP53*-KD cells (Fig. 4B, C). This indicated that, while *TP53* mRNA level is upregulated in D458 MB cells following hypoxia, wtp53 does not play a role in mediating cell death under these conditions (Fig. 4B, C). As shown by the IC_50_ values, the response of *TP53*-KD D458 cells to simvastatin under hypoxia was comparable to that of their respective scramble-transfected controls (i.e. still wtp53) (Fig. 4C). As shown by the IC_50_ values, DAOY displayed a similar response to simvastatin under normoxia or hypoxia (Fig. 4B). Control and *TP53*-KD DAOY cells also exhibited comparable sensitivity to simvastatin under both conditions (Fig. 4B). Collectively, the results indicate that severe hypoxia fails to increase the sensitivity of MB cells to simvastatin, independently of p53 status.

### DNA damage does not affect MB response to simvastatin irrespective of p53 status

X-ray radiation has long been employed in research and clinical settings to induce DNA double-strand breaks and trigger DNA damage response pathways. DNA damage leads to the post-translational modification and stabilisation of p53, enhancing its function as a transcription factor to induce cell cycle arrest, DNA repair pathways, and apoptosis to prevent the spread of damaged DNA and maintain genome integrity [38, 40]. Given the standard use of full craniospinal irradiation in treating high-risk MB patients, we next explored the potential of simvastatin to act as an adjuvant to radiation therapy and how p53 status might modulate MB sensitivity to this combined treatment.

We began by evaluating whether clinically relevant doses of X-ray irradiation (5 Gy) could affect p53 protein levels in DAOY and D458 cells. As expected, we observed an increase in p53 protein levels upon ionising radiation in D458 cells that retained wtp53 but no change in DAOY cells harbouring mutp53 (Fig. 4D, Supplementary Fig. 3A).

Next, we examined whether prior X-ray irradiation (5 Gy) could affect MB cell sensitivity to various doses of simvastatin (0–100 µM). Pre-irradiation elicited little cytotoxicity in D458 in both scramble-transfected and *TP53*-KD D458 cells. However, such cytotoxicity was not additive to the effect of simvastatin (Fig. 4E). Conversely, prior irradiation did not significantly affect DAOY cells viability and did not alter simvastatin-induced cytotoxicity (Fig. 4F). Moreover, *TP53-KD* had no impact upon their response to these combined treatments (Fig. 4E, F). Together, these data indicate that *TP53* depletion combined with irradiation did not show synergistic effect with simvastatin-induced cytotoxicity in MB cells, regardless of p53 mutational status.

### DNA hypomethylating agents affect MB cell viability in a mutp53-dependent manner, but do not have additive effects with simvastatin

Alteration of the epigenetic landscape such as aberrant DNA methylation, drives to the reshaping of transcriptional program which in turn contributes to tumorigenesis [45, 46]. Efforts to reverse these aberrant epigenetic modifications has led to the development of inhibitors directed toward the enzyme responsible for DNA methylation, DNA methyltransferase-1 (DNMT1) [47]. Two such inhibitors of DNMT1 (DNMTIs), decitabine and azacitidine, have demonstrated great therapeutic potential and are currently in use for treatment of myelodysplastic disorders, chronic myelomonocytic leukaemia and acute myeloid leukaemia (AML) [47, 48].

**Fig. 5:**
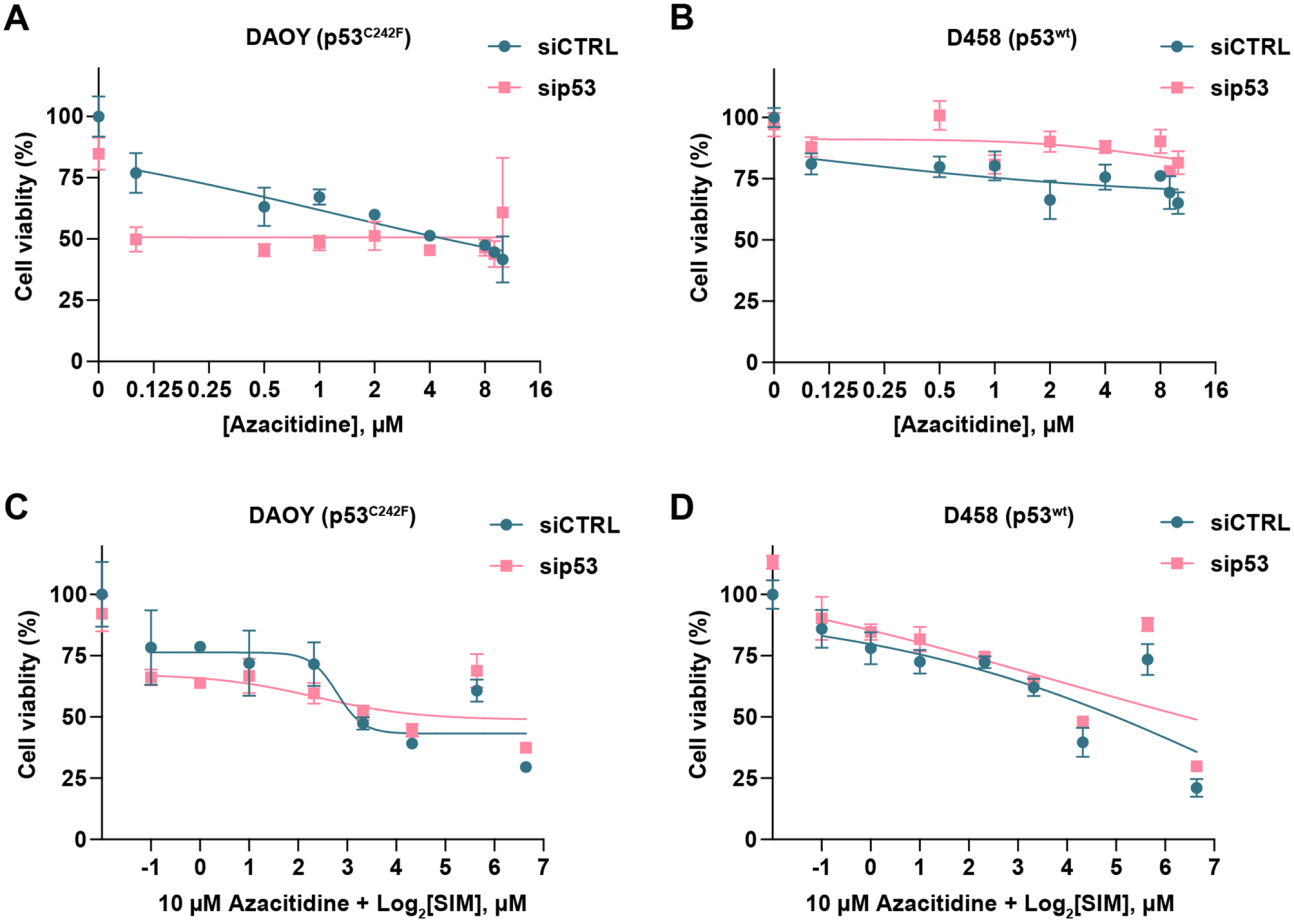
Response to DNA hypomethylating in MB cells. **A, B**) Dose-response curves were determined using the MTT assay in DAOY (**A**) and D458 (**B**) cells following azacitidine treatment, with or without *TP53* silencing. MB cells were treated in the presence of increasing concentrations of azacitidine (0.1, 0,5, 1, 2, 4, 8, 9 and 10 µM) for 72 hours. All values were normalised to the siCTRL untreated group, which was set to 100%. **C, D**) Dose-response curves were determined using the MTT assay in DAOY (**C**) and D458 (**D**) cells following combination treatment. MB cells were treated in the presence of increasing concentrations of simvastatin (0.5, 1, 2, 5, 10, 20, 50 and 100 µM) combined with 10 μM azacitidine for 72 hours. All values were normalised to the siCTRL untreated group, which was set to 100%. Curve fitting and IC_50_ values were evaluated using GraphPad prism 10 with a nonlinear regression model ([inhibitor] vs. response, variable slope, four parameters) (*n* = 3).

MBs, particularly the SHH subgroup, are characterised by extensive epigenetic remodelling and tumour suppressor gene silencing [49, 50], however hypomethylating agents remain relatively unstudied in brain tumours, hence we decided to investigate whether azacitidine, alone or in combination with simvastatin, could represent a potential new therapeutic strategy for these tumours. Depletion of mutp53 protein enhanced azacitidine sensitivity in DAOY cells, whereas D458 cells appeared generally resistant to Azacitidine (Fig. 5A, B). Notably, the effect of azacitidine in DAOY did not appear to be dose-dependent, with similar cytotoxicity observed across concentrations range tested (Fig. 5A). Combined administration of azacitidine (10 µM) and simvastatin (0–100 µM) in DAOY cells failed to enhance MB cytotoxicity beyond that induced by either agent alone, and even appeared to slightly dampen the response to simvastatin (Fig. 5C). In D458 cells, Azacitidine did not alter the profile of response to simvastatin (Fig. 5D). All the responses were not significantly altered by p53 status (Fig. 5). Together, these data suggest that D458 cells, which represent Group 3 MB, are more sensitive to treatment with DNMTIs or hypomethylating agents than DAOY cells, which represent SHH-MB Group.

## Discussion

In this study, we examined whether *TP53* status modulates MB sensitivity to inhibition of the MVP by simvastatin, and whether combining simvastatin with radiotherapy or epigenetic therapy enhances therapeutic efficacy. Mutp53 proteins are known to acquire oncogenic gain-of-function properties, including metabolic reprogramming mediated in part through upregulation of the MVP, which supports cholesterol and isoprenoid biosynthesis [21, 51]. Given that *TP53* is mutated in up to 30% of SHH-subgroup MB and is associated with poor clinical outcome, we hypothesised that mutp53 may confer increased dependence on the MVP, thereby sensitising tumours to statin treatment.

Although mutp53 MB cell lines exhibited greater sensitivity to simvastatin compared with wtp53 cells, depletion of p53 protein by *TP53* knockdown did not significantly alter simvastatin-induced cytotoxicity in any model examined. Moreover, *TP53* silencing had no effect on the expression of key MVP enzymes, including HMGCR, FDPS, MVK, and MVD, indicating that MVP regulation in MB cells occurs independently of p53 status.

Under conditions of severe hypoxia, a common feature of solid tumours [43], including MB, p53 depletion did not influence cell viability or sensitivity to simvastatin. Similarly, irradiation increased p53 protein levels exclusively in wtp53 cells and produced a modest reduction in viability; however, neither radiosensitivity nor the response to combined simvastatin treatment was affected by *TP53* knockdown. In addition, co-treatment with the DNMT inhibitor azacitidine failed to enhance simvastatin-mediated cytotoxicity.

Collectively, these findings indicate that MVP activity and the therapeutic response to simvastatin in MB are largely independent of p53 status. Future studies should therefore investigate alternative regulators of the MVP, including MYC family members or mTOR signalling, to better define mechanisms that may optimise statin-based therapeutic strategies in MB.

## Materials and methods

### Cell culture

MB cell lines (DAOY, UW228-2, and D458) were cultured using methods previously described by Merve et al. [52]. DAOY and UW228-2 cells are a model of SHH subgroup MB while D458 represents a model of the G3 and G4-MB subgroups. Each MB cell line was cultured in Dulbecco’s Modified Eagle’s Medium (DMEM; Gibco^TM^, US) with GlutaMAX^TM^, supplemented with 1% MEM Non-Essential Amino Acids Solution, 10% foetal bovine serum (FBS) and 1% penicillin streptomycin-glutamine (Gibco™, US). Cells were incubated at 37 °C and 5% CO_2_ and subcultured every 2–3 days. DAOY and UW228-2 cells were grown as an adhesive monolayer, while D458 cells were grown as semi-adherent cultures. DAOY and UW228-2 cell lines were obtained from ATCC, D458 cell line was obtained from Dr Silvia Marino, Queen Mary University of London.

### Chemicals

Simvastatin powder (20 mg) purchased from US Pharmacopeia (USP, US) was reconstituted in 2.5 mL pure EtOH with 750 μL NaOH and heated at 50 °C for 2 hours to activate the prodrug. The pH was adjusted to 7 with 1 M HCl and then made up to 5 mL with EtOH to make a stock concentration of 1.1945 mM.

DNA hypomethylating agent azacitidine (#A3656-5 mg, 5-aza-2’deoxycitidine, Sigma, US) was resuspended in DMSO at concentration of 10 mM and used at the indicated concentrations.

### siRNA transfection

MB cell lines were transfected with *TP53*-targeting siRNA (sip53, #4390825, ID: s605., ThermoFisher, US) or scrambled control siRNA (siCTRL, #4390844, Negative control #1, ThermoFisher, US) using Lipofectamine RNAiMAX reagent (#13,778,150, Invitrogen, US) via either forward transfection on 6- or 10-cm dishes or reverse transfection on 96-well plates according to the manufacturer’s instructions. Cells were harvested 24 hours post-transfection.

### RNA extraction and real-time quantitative PCR (RT-qPCR)

Total RNA was isolated from MB cell line pellets for *TP53* silencing checks and MVP gene expression analyses using the Qiagen RNeasy mini kit according to manufacturer’s protocols. cDNA was reverse transcribed from extracted Total RNA using a RevertAid first strand cDNA synthesis kit (ThermoFisher, US) and a C1000 Touch Thermal Cycler (Bio-Rad, US) following the manufacturer’s protocols. RT-qPCR was subsequently conducted using SYBR Green master mix and human-specific primers corresponding to *HMGCR*, *FDPS*, *MVK*, *MVD*, *TP53*, *CDKN1A*, and *TAp73* on a QuantStudio™ Real-Time PCR System (ThermoFisher, US) according to manufacturer’s protocols. The TATA-box Binding Protein (*TBP*) was used as a housekeeping gene for normalisation. Primer sequences are listed in Supplementary Table 1. Relative expression levels were calculated using the 2^-ΔΔCt^ method.

### Cell viability assays

Cell viability assays were carried out using an MTT (3-(4,5-dimethylthiazol-2-yl)-2,5-diphenyltetrazolium bromide) assay kit (#ab211091, Abcam, UK). For cytotoxicity assays involving simvastatin alone, MB cell lines were seeded in triplicate upon clear-bottom 96-well plates (Greiner AG, Austria) at 5000 cells/well. After 24 hours, cells were then transfected with sip53 or siCTRL overnight. The following day, cells were treated with increasing concentrations of simvastatin (0–100 µM) for 48 h. For cytotoxicity assays involving azacitidine alone (0–10 µM) and in combination with simvastatin (0–100 µM simvastatin and 10 µM azacitidine fixed concentration), DAOY and D458 cells were seeded into 96-well plates following a reverse transfection protocol to align with the maximum transient transfection time of 72 h silencing.

Following treatment (48 or 72 hours), media was removed and replaced with MTT solution pre-diluted in complete DMEM to a final concentration of 0.45 mg/mL and incubated for 2–4 hours. Once formazan crystals were present, media was removed and crystals dissolved using DMSO before absorbance was read at 570 nm using a Tecan infinite 200 microplate reader (Tecan, Switzerland).

For DNA damage-simvastatin response assays, MB cell lines were seeded in 96-well plates following the same reverse transfection protocol, and administered X-Ray irradiation (5 Gy) using the XRad 225 XL apparatus. 24 hours post-irradiation, cells were then treated with simvastatin (0–100 µM). Non-irradiated plates were also treated with simvastatin in parallel to act as a comparative control. After 48 hours, cell viability was measured using the MTT assay, following the same steps described above.

For hypoxia-simvastatin response assays, MB cells were seeded and reverse transfected in 96-well plates. The following day, the cells were transferred to a hypoxic (1% O_2_) incubator. After a 24-hour equilibration period, hypoxic cells were treated with simvastatin (0–100 µM) diluted in fresh DMEM. Plates cultured in normoxic conditions were also treated with simvastatin in parallel to act as a comparative control. After 48 hours, cell viability was measured using the MTT assay. Hypoxia experiments (including addition of MTT solution) were carried out within a Sci-Tive hypoxia workstation to ensure that cells remained under 1% O_2_ and were not exposed to normoxia prior to the experimental endpoint.

### Cell cycle analysis by flow cytometry

Cell cycle progression of p53 proficient- (siCTRL) and deficient- (sip53) MB cell lines treated with 5 µM simvastatin for 24 or 48 hours was assessed via flow cytometry. After simvastatin treatment, cells were harvested by trypsinization. The resulting cell pellet was then fixed and permeabilized with 4:1 of ice-cold methanol:acetone solution for a minimum of 1 hour at 4 °C. Following fixation, each sample was washed with PBS 1x. The final sample pellet was resuspended and incubated with 20 μg/mL of RNAse A and then labelled with propidium iodide solution (100 μg/mL) for 30 minutes in the dark or overnight. Cell cycle profile was acquired using a BD FACS Canto system (BD Biosciences, US) with BD FACSDiva software and analysed with FlowJo flow cytometry software.

### Immunoblotting

Cells were collected and lysed in RIPA buffer supplemented with protease and phosphatase inhibitor cocktail. Proteins were extracted following sonication and centrifugation at 4 °C. After protein quantification using the Bradford assay (#5000006, Biorad, US), equal amounts of protein were diluted in distilled water and loading dye, mixed thoroughly, and boiled at 95 °C for 10 min.

Proteins were resolved on a 10% Sodium Dodecyl Sulfate polyacrylamide gel (SDS-PAGE) to detect the expression levels of p53 (sc-126, Santa Cruz Biotechnology, US) and GAPDH (#60,004, Proteintech Group Inc, US). Proteins were then transferred onto polyvinylidene difluoride (PVDF) membrane via Trans-Blot Turbo Transfer System (Bio-Rad Laboratories, US). Membranes were blocked with 5% non-fat milk in phosphate buffered saline with Tween-20 (PBST) at room temperature (RT) for 1 h and then incubated with primary antibodies overnight at 4 °C. Membranes were washed 3 times with PBST and then incubated with the appropriate HRP-conjugated secondary antibodies at RT for 2 h. Immunoblots were imaged via ImageQuant 800 Western blot imaging systems (Amersham, UK). Molecular weight was indicated via a broad range protein ladder (#26623, Thermo Fisher Scientific, US).

### Quantification and statistical analysis

GraphPad Prism 10 was used for the graphical representation and calculation of the statistical tests. All results are expressed as mean values ± SEM of at least three independent experiments. The analysis of variance (one-way ANOVA) was used to assess significant differences between results. *p*-values of <0.05 were considered statistically significant.

## Electronic supplementary material

Below is the link to the electronic supplementary material.

Supplementary material 1

Supplementary material 2

Supplementary material 3

## Data Availability

The data will be available upon request.

## References

[1] Onyije FM, Dolatkhah R, Olsson A, Bouaoun L, Deltour I, Erdmann F, et al. Risk factors for childhood brain tumours: a systematic review and meta-analysis of observational studies from 1976 to 2022. Cancer Epidemiol. 2024;88:102510. 38056243 10.1016/j.canep.2023.102510 PMC10835339

[2] Archer TC, Mahoney EL, Pomeroy SL. Medulloblastoma: molecular classification-based personal therapeutics. Neurotherapeutics. 2017;14(2):265–73. 28386677 10.1007/s13311-017-0526-y PMC5398996

[3] Choi JY. Medulloblastoma: current perspectives and recent advances. Brain Tumor Res Treat. 2023;11(1):28–38. 36762806 10.14791/btrt.2022.0046 PMC9911713

[4] Osuna-Marco MP, Martín-López LI, M TÁ, López-Ibor B. Questions and answers in the management of children with medulloblastoma over the time. How did we get here? A systematic review. Front Oncol. 2023;13:1229853. 37456257 10.3389/fonc.2023.1229853 PMC10340518

[5] Menyhárt O, Győrffy B. Principles of tumorigenesis and emerging molecular drivers of SHH-activated medulloblastomas. Ann Clin Transl Neurol. 2019;6(5):990–1005. 31139698 10.1002/acn3.762 PMC6529984

[6] Phoenix TN, Patmore DM, Boop S, Boulos N, Jacus MO, Patel YT, et al. Medulloblastoma genotype dictates blood brain barrier phenotype. Cancer Cell. 2016;29(4):508–22. 27050100 10.1016/j.ccell.2016.03.002 PMC4829447

[7] Kool M, Korshunov A, Remke M, Jones DT, Schlanstein M, Northcott PA, et al. Molecular subgroups of medulloblastoma: an international meta-analysis of transcriptome, genetic aberrations, and clinical data of WNT, SHH, group 3, and group 4 medulloblastomas. Acta Neuropathol. 2012;123(4):473–84. 22358457 10.1007/s00401-012-0958-8 PMC3306778

[8] Fu Y, Zou T, Shen X, Nelson PJ, Li J, Wu C, et al. Lipid metabolism in cancer progression and therapeutic strategies. Med Comm (2020), 2(1):27–59. 34766135 10.1002/mco2.27 PMC8491217

[9] Dai C-L, Qiu Z-Y, Wang A-Q, Yan S, Zhang L-J, Luan X. Targeting cholesterol metabolism: a promising therapy strategy for cancer. Acta Pharmacologica Sin. 2025;46(8):2093–104. 10.1038/s41401-025-01531-9 PMC12274607 40133625

[10] Jin U, Park SJ, Park SM. Cholesterol metabolism in the brain and its association with Parkinson’s disease. Exp Neurobiol. 2019;28(5):554–67. 31698548 10.5607/en.2019.28.5.554 PMC6844833

[11] Parri M, Chiarugi P. Rac and Rho GTPases in cancer cell motility control. Cell Commun Signal. 2010;8:23. 20822528 10.1186/1478-811X-8-23 PMC2941746

[12] Berndt N, Hamilton AD, Sebti SM. Targeting protein prenylation for cancer therapy. Nat Rev Cancer. 2011;11(11):775–91. 22020205 10.1038/nrc3151 PMC4037130

[13] Maldonado MDM, Dharmawardhane S. Targeting Rac and Cdc42 GTPases in cancer. Cancer Res. 2018;78(12):3101–11. 29858187 10.1158/0008-5472.CAN-18-0619 PMC6004249

[14] Lasunción MA, Martínez-Botas J, Martín-Sánchez C, Busto R, Gómez-Coronado D. Cell cycle dependence on the mevalonate pathway: role of cholesterol and non-sterol isoprenoids. Biochemical Pharmacol. 2022;196:114623. 10.1016/j.bcp.2021.114623 34052188

[15] Radhakrishnan A, Goldstein JL, McDonald JG, Brown MS. Switch-like control of SREBP-2 transport triggered by small changes in ER cholesterol: a delicate balance. Cell Metab. 2008;8(6):512–21. 19041766 10.1016/j.cmet.2008.10.008 PMC2652870

[16] Loughran RM, Emerling BM. Mechanistic roles of mutant p53 governing lipid metabolism. Adv Biol Regul. 2022;83:100839. 34840111 10.1016/j.jbior.2021.100839 PMC8858851

[17] Labuschagne CF, Zani F, Vousden KH. Control of metabolism by p53 - cancer and beyond. Biochim Et Biophys Acta (BBA) - Rev Cancer. 2018;1870(1):32–42. 10.1016/j.bbcan.2018.06.001 PMC6102416 29883595

[18] Janic A, Abad E, Amelio I. Decoding p53 tumor suppression: a crosstalk between genomic stability and epigenetic control? Cell Death Differ. 2025;32(1):1–8. 38379088 10.1038/s41418-024-01259-9 PMC11742645

[19] Panatta E, Butera A, Mammarella E, Pitolli C, Mauriello A, Leist M, et al. Metabolic regulation by p53 prevents R-loop-associated genomic instability. Cell Rep. 2022;41(5):111568. 36323249 10.1016/j.celrep.2022.111568

[20] Boutelle AM, Mabene AR, Yao D, Xu H, Wang M, Tang YJ, et al. Integrative multiomic approaches reveal ZMAT3 and p21 as conserved hubs in the p53 tumor suppression network. Cell Death Differ. 2025;32(11):1954–69. 40263541 10.1038/s41418-025-01513-8 PMC12572384

[21] Butera A, Amelio I. Deciphering the significance of p53 mutant proteins. Trends Cell Biol. 2025;35(3):258–68. 38960851 10.1016/j.tcb.2024.06.003

[22] Attardi LD, Boutelle AM. Targeting p53 gain-of-function activity in cancer therapy: a cautionary tale. Cell Death Differ. 2024;31(2):133–35. 38151545 10.1038/s41418-023-01253-7 PMC10850540

[23] Pitolli C, Wang Y, Mancini M, Shi Y, Melino G, Amelio I. Do mutations turn p53 into an oncogene? Int J Mol Sci. 2019;20(24). 10.3390/ijms20246241 PMC6940991 31835684

[24] Caporali S, Butera A, Ruzza A, Zampieri C, Bantula M, Scharsich S, et al. Selective metabolic regulations by p53 mutant variants in pancreatic cancer. J Exp Clin Cancer Res. 2024;43(1):310. 39587609 10.1186/s13046-024-03232-3 PMC11590503

[25] Cotton K, Comer C, Caporali S, Butera A, Gurres S, Capradossi F, et al. Lipidome atlas of p53 mutant variants in pancreatic cancer. Biol Direct. 2025;20(1):51. 40217553 10.1186/s13062-025-00635-w PMC11992884

[26] Freed-Pastor WA, Mizuno H, Zhao X, Langerød A, Moon SH, Rodriguez-Barrueco R, et al. Mutant p53 disrupts mammary tissue architecture via the mevalonate pathway. Cell. 2012;148(1–2):244–58. 22265415 10.1016/j.cell.2011.12.017 PMC3511889

[27] Moon SH, Huang CH, Houlihan SL, Regunath K, Freed-Pastor WA, JPt M, et al. p53 represses the mevalonate pathway to mediate tumor suppression. Cell. 2019;176(3):564–80 e19. 30580964 10.1016/j.cell.2018.11.011 PMC6483089

[28] Oni TE, Biffi G, Baker LA, Hao Y, Tonelli C, Somerville TDD, et al. SOAT1 promotes mevalonate pathway dependency in pancreatic cancer. J Exp Med. 2020;217(9). 10.1084/jem.20192389 PMC7478739 32633781

[29] Parrales A, Ranjan A, Iyer SV, Padhye S, Weir SJ, Roy A, et al. DNAJA1 controls the fate of misfolded mutant p53 through the mevalonate pathway. Nat Cell Biol. 2016;18(11):1233–43. 27775703 10.1038/ncb3427 PMC5340314

[30] Parrales A, Thoenen E, Iwakuma T. The interplay between mutant p53 and the mevalonate pathway. Cell Death Differ. 2018;25(3):460–70. 29238070 10.1038/s41418-017-0026-y PMC5864191

[31] Ingallina E, Sorrentino G, Bertolio R, Lisek K, Zannini A, Azzolin L, et al. Mechanical cues control mutant p53 stability through a mevalonate-RhoA axis. Nat Cell Biol. 2018;20(1):28–35. 29255172 10.1038/s41556-017-0009-8 PMC6179142

[32] Comer C, Cotton K, Edwards C, Dai X, Badodi S, Buccafusca R, et al. Simvastatin suppresses spinal cord metastasis of medulloblastoma at clinically significant doses. Cell Death Dis. 2025;16(1):527. 40664675 10.1038/s41419-025-07829-0 PMC12263873

[33] Zhukova N, Ramaswamy V, Remke M, Pfaff E, Shih DJ, Martin DC, et al. Subgroup-specific prognostic implications of TP53 mutation in medulloblastoma. J Clin Oncol. 2013;31(23):2927–35. 23835706 10.1200/JCO.2012.48.5052 PMC4878050

[34] Eibl RH, Schneemann M. Medulloblastoma: from TP53 mutations to molecular classification and liquid biopsy. Biol (Basel). 2023;12(2). 10.3390/biology12020267 PMC9952923 36829544

[35] Künkele A, De Preter K, Heukamp L, Thor T, Pajtler KW, Hartmann W, et al. Pharmacological activation of the p53 pathway by nutlin-3 exerts anti-tumoral effects in medulloblastomas. Neuro Oncol. 2012;14(7):859–69. 22591662 10.1093/neuonc/nos115 PMC3379802

[36] Guerra B, Recio C, Aranda-Tavío H, Guerra-Rodríguez M, García-Castellano JM, Fernández-Pérez L. The mevalonate pathway, a metabolic Target in cancer therapy. Front Oncol. 2021;11:626971. 33718197 10.3389/fonc.2021.626971 PMC7947625

[37] Niklison-Chirou MV, Erngren I, Engskog M, Haglöf J, Picard D, Remke M, et al. TAp73 is a marker of glutamine addiction in medulloblastoma. Genes Dev. 2017;31(17):1738–53. 28971956 10.1101/gad.302349.117 PMC5666673

[38] Abuetabh Y, Wu HH, Chai C, Al Yousef H, Persad S, Sergi CM, et al. DNA damage response revisited: the p53 family and its regulators provide endless cancer therapy opportunities. Exp Mol Med. 2022;54(10):1658–69. 36207426 10.1038/s12276-022-00863-4 PMC9636249

[39] Amelio I, Antonov AA, Catani MV, Massoud R, Bernassola F, Knight RA, et al. TAp73 promotes anabolism. Oncotarget. 2014;5(24):12820–934. 25514460 10.18632/oncotarget.2667 PMC4350352

[40] Wang H, Guo M, Wei H, Chen Y. Targeting p53 pathways: mechanisms, structures, and advances in therapy. Signal Transduct Target Ther. 2023;8(1):92. 36859359 10.1038/s41392-023-01347-1 PMC9977964

[41] Pallottini V, Guantario B, Martini C, Totta P, Filippi I, Carraro F, et al. Regulation of HMG-CoA reductase expression by hypoxia. J Cell Biochem. 2008;104(3):701–09. 18459144 10.1002/jcb.21757

[42] Amelio I, Mancini M, Petrova V, Cairns RA, Vikhreva P, Nicolai S, et al. p53 mutants cooperate with HIF-1 in transcriptional regulation of extracellular matrix components to promote tumor progression. Proc Natl Acad Sci U S A. 2018;115(46):E10869–78. 10.1073/pnas.1808314115 PMC6243248 30381462

[43] Petrova V, Annicchiarico-Petruzzelli M, Melino G, Amelio I. The hypoxic tumour microenvironment. Oncogenesis. 2018;7(1):10. 29362402 10.1038/s41389-017-0011-9 PMC5833859

[44] Amelio I, Melino G. The p53 family and the hypoxia-inducible factors (HIFs): determinants of cancer progression. Trends Biochem Sci. 2015;40(8):425–34. 26032560 10.1016/j.tibs.2015.04.007

[45] Sato T, Issa JJ, Kropf P. DNA hypomethylating drugs in cancer therapy. Cold Spring Harb Perspect Med. 2017;7(5). 10.1101/cshperspect.a026948 PMC5411681 28159832

[46] Butera A, Melino G, Amelio I. Epigenetic “drivers” of cancer. J Mol Biol. 2021;433(15):167094. 34119490 10.1016/j.jmb.2021.167094

[47] Derissen EJ, Beijnen JH, Schellens JH. Concise drug review: azacitidine and decitabine. Oncologist. 2013;18(5):619–24. 23671007 10.1634/theoncologist.2012-0465 PMC3662854

[48] Baylin SB, Jones PA. A decade of exploring the cancer epigenome - biological and translational implications. Nat Rev Cancer. 2011;11(10):726–34. 21941284 10.1038/nrc3130 PMC3307543

[49] Yi J, Wu J. Epigenetic regulation in medulloblastoma. Mol Cell Neurosci. 2018;87:65–76. 29269116 10.1016/j.mcn.2017.09.003 PMC5828953

[50] Bonifacio-Mundaca J, Casavilca-Zambrano S, Desterke C, Casafont Í, Mata-Garrido J. Deciphering medulloblastoma: epigenetic and metabolic changes driving tumorigenesis and treatment outcomes. Biomedicines. 2025;13(8). 10.3390/biomedicines13081898 PMC12383691 40868156

[51] Freed-Pastor WA, Prives C. Mutant p53: one name, many proteins. Genes Dev. 2012;26(12):1268–86. 22713868 10.1101/gad.190678.112 PMC3387655

[52] Merve A, Dubuc AM, Zhang X, Remke M, Baxter PA, Li XN, et al. Polycomb group gene BMI1 controls invasion of medulloblastoma cells and inhibits BMP-regulated cell adhesion. Acta Neuropathol Commun. 2014;2:10. 24460684 10.1186/2051-5960-2-10 PMC3928978

