# Supplementary material for: Medulloblastoma response to mevalonate pathway inhibition is independent of p53 status
Source: Biol Direct. 2026 Apr 1;21:42. doi: 10.1186/s13062-026-00765-9 (PMC13063774; doi:10.1186/s13062-026-00765-9)

Supplementary Figure 1

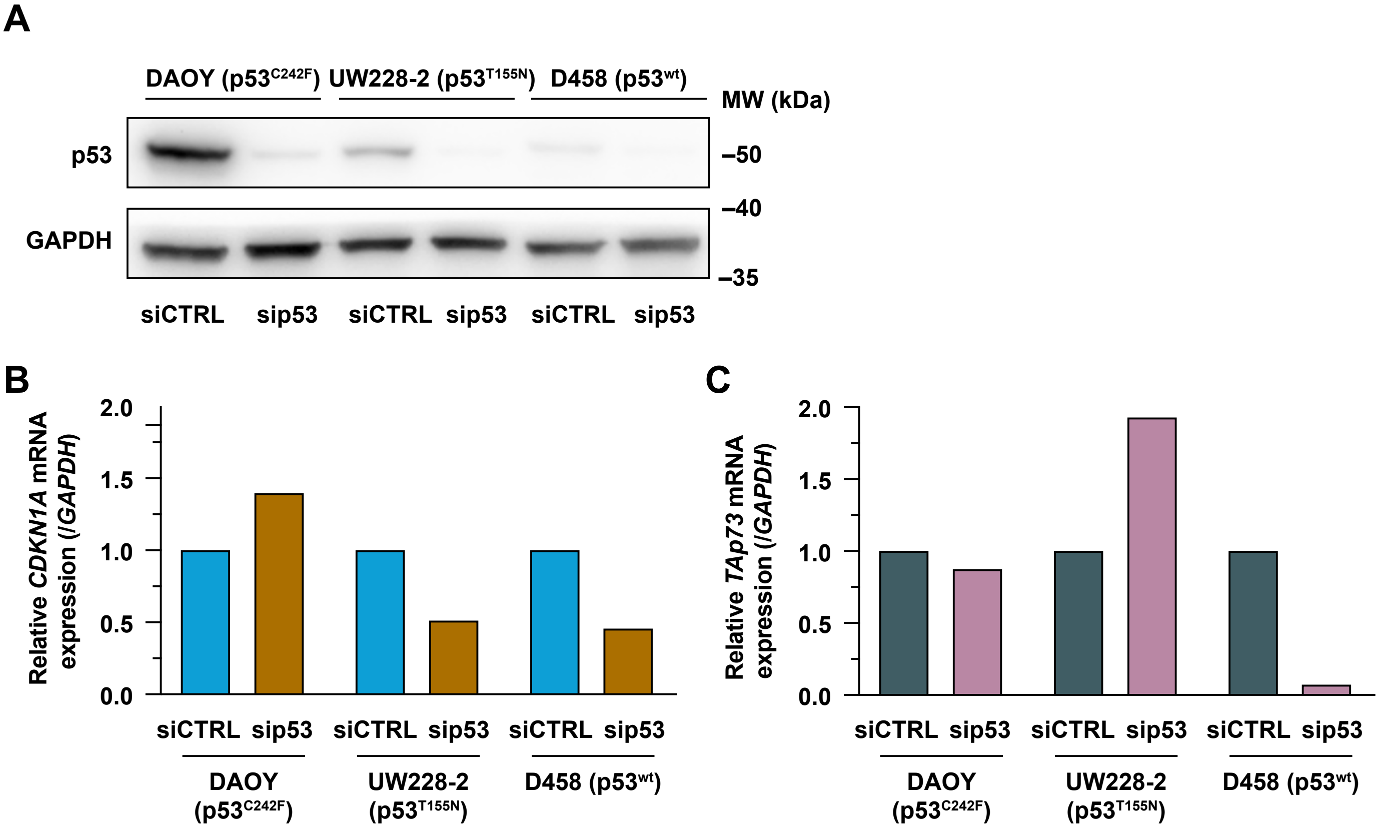

Supplementary Figure 2

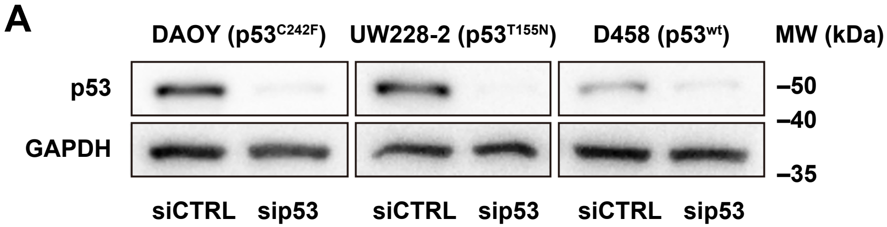

Supplementary Figure 3

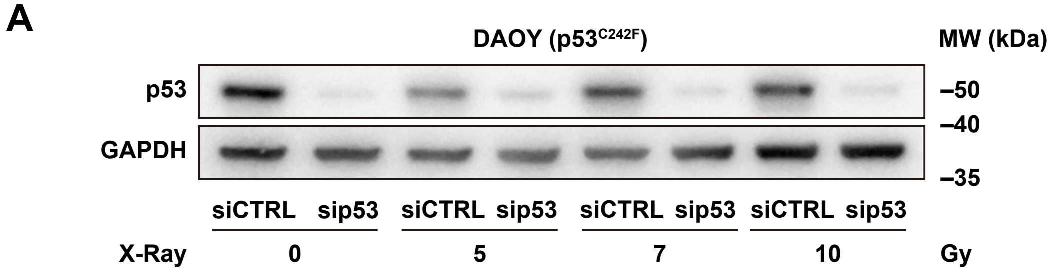

Supplementary Figure 4

Figure 1B

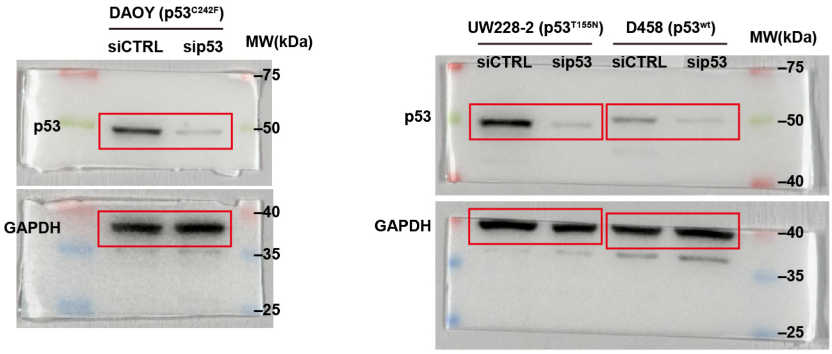

Figure 3B

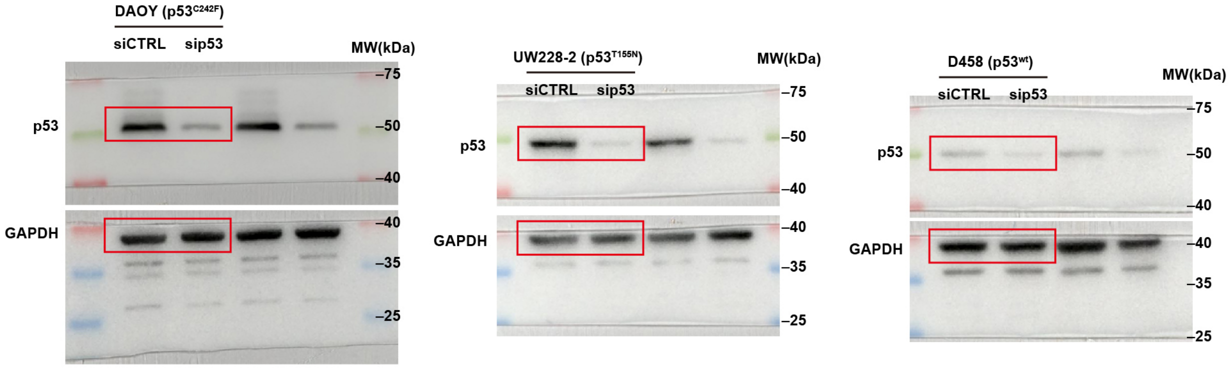

Figure 4D

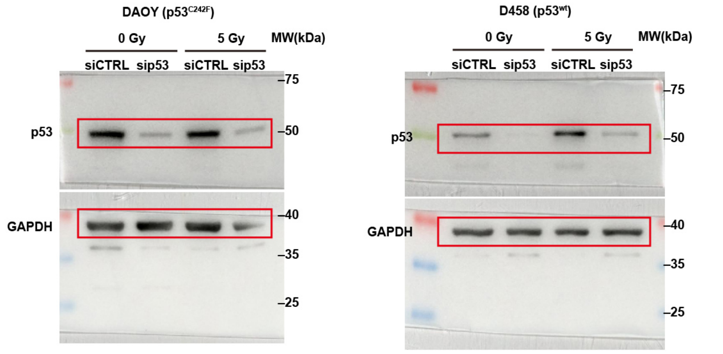

Supplementary Figure 1A

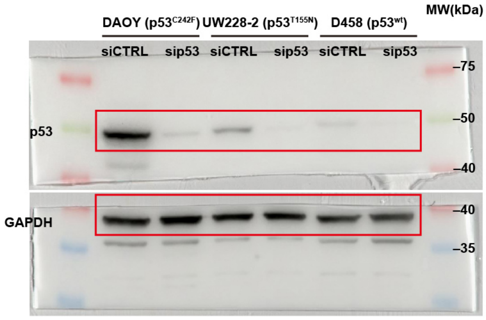

Supplementary Figure 2A

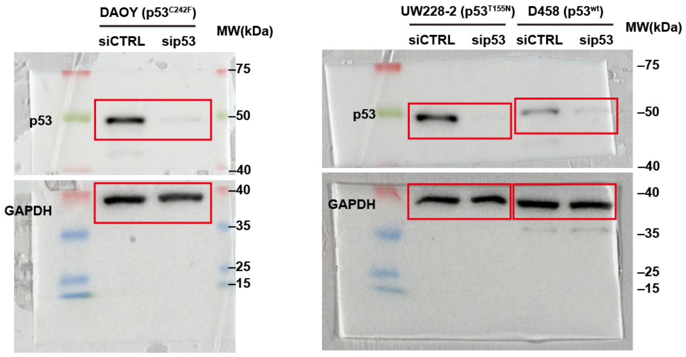

Supplementary Figure 3A

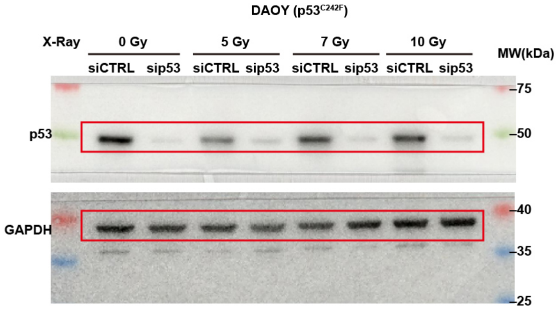

Supplement: Supplementary file 1 — Supplementary material 1 [file 13062_2026_765_MOESM1_ESM.pdf]
