# Supplementary material for: Medulloblastoma response to mevalonate pathway inhibition is independent of p53 status
Source: Biol Direct. 2026 Apr 1;21:42. doi: 10.1186/s13062-026-00765-9 (PMC13063774; doi:10.1186/s13062-026-00765-9)
Supplement: Supplementary file 2 — Supplementary material 2 [file 13062_2026_765_MOESM2_ESM.docx]

**Supplementary Figure:**

**Supplementary figure 1. Immunoblot analysis of *TP53*-KD efficiency across MB cell lines shows marked reduction in p53 expression. A**) Representative immunoblot showing *TP53* silencing in DAOY, UW228-2 and D458 cells. **B**) RT-qPCR analyses of *CDKN1A* mRNA expression in MB cell lines following *TP53* KD. **C**) Representative RT-qPCR analyses of *TAp73* mRNA levels in DAOY, UW228-2 and D458 cells.

**Supplementary figure 2. Repeated immunoblots highlighting silencing efficacy of *TP53* with lipofectamine reagent. A)** *TP53* silencing of DAOY, UW228-2 and D458 MB cell lines following transient transfection with siRNA.

**Supplementary figure 3. Immunoblot analysis of p53 response to X-ray irradiation and *TP53* silencing efficiency. A)** Representative immunoblot of p53 protein level in DAOY cells following X-ray irradiation from 0–10 Gy.

**Supplementary figure 4. Uncropped immunoblots.**
