# Supplementary material for: Medulloblastoma response to mevalonate pathway inhibition is independent of p53 status
Source: Biol Direct. 2026 Apr 1;21:42. doi: 10.1186/s13062-026-00765-9 (PMC13063774; doi:10.1186/s13062-026-00765-9)
Supplement: Supplementary file 3 — Supplementary material 3 [file 13062_2026_765_MOESM3_ESM.docx]

**Supplementary table 1: List of human forward and reverse primers used in this study.** List of human forward (FWD) and reverse (REV) primer sequences for key MVP enzymes: *HMGCR*, *MVK*, *MVD* and *FDPS*, in addition to *TP53*, *CDKN1A*, *TAp73* and *TBP* (loading control).

| **Gene name** | **FWD** | **REV** |
| --- | --- | --- |
| 3-hydroxy-3-methylglutaryl-CoA reductase (*HMGCR*) | GGCCCAGTTGTGCGTCTT | CGAGCCAGGCTTTCACTTCT |
| Mevalonate kinase (MVK) | TGGACCTCAGCTTACCCAACA | GACTGAAGCCTGGCCACATC |
| Mevalonate decarboxylase (MVD) | TGAACTCCGCGTGCTCATC | CGGTACTGCCTGTCAGCTTCT |
| Farnesyl diphosphate synthase (*FDPS*) | CTTCCTATAGCTGCAGCCATGTAC | GCATTGGCGTGCTCCTTCT |
| Tumour suppressor protein p53 (*TP53*) | CCCAAGCAATGGATGATTTGA | GGCATTCTGGGAGCTTCATCT |
| p21 / Cyclin-dependent kinase inhibitor 1 (*CDKN1A*) | TGGGGATGTCCGTCAGAAC | GGCGTTTGGAGTGGTAGAAATC |
| Tumour protein p73 (*TAp73*) | CAGACAGCACCTACTTCGA | CCGCCCACCACCTCATTA |
| TATA-box binding protein (*TBP*) | TCAAACCCAGAATTGTTCTCCTTAT | TCAAACCCAGAATTGTTCTCCTTAT |
